# Assessment of bidirectional relationships between circulating cytokines and periodontitis: Insights from a mendelian randomization analysis

**DOI:** 10.3389/fgene.2023.1124638

**Published:** 2023-01-30

**Authors:** Shi-Jia Huang, Rong Li, Shuo Xu, Yuan Liu, Sheng-Hui Li, Sheng-Zhong Duan

**Affiliations:** ^1^ Laboratory of Oral Microbiota and Systemic Diseases, Shanghai Ninth People’s Hospital, College of Stomatology, Shanghai Jiao Tong University School of Medicine, Shanghai, China; ^2^ Shanghai Key Laboratory of Stomatology, National Center for Stomatology, National Clinical Research Center for Oral Diseases, Shanghai, China; ^3^ MOE-Shanghai Key Laboratory of Children’s Environmental Health, School of Public Health, Shanghai Jiao Tong University School of Medicine, Shanghai, China

**Keywords:** periodontitis, cytokines, IL9, IL17, mendelian randomization

## Abstract

**Background:** The purpose of this Mendelian randomization (MR) study was to assess the causal relationship between circulating cytokines and periodontitis.

**Materials and methods:** Based on the aggregated statistics of the largest publicly available genome-wide association study (GWAS), we applied a bidirectional two-sample MR. MR analyses were conducted using Inverse variance weighted (IVW), Robust Adjusted Profile Score (RAPS), Maximum likelihood (ML), Weighted median and MR-Egger, and results obtained from IVW served as the primary outcome. Cochran Q test was used to test the heterogeneity. MR-Egger intercept test and MR polymorphism residual and outlier test (MR-PRESSO) were used for polymorphism analysis. Leave-one-out sensitivity and funnel plots were used for sensitivity analysis.

**Results:** The IVW method indicated that interleukin 9 (IL9) had a positive causal relationship with periodontitis [odds ratio (OR) = 1.199, 95% confidence interval (CI) = 1.049–1.372, *p* = 0.008], and interleukin 17 (IL17) had a negative causal relationship with periodontitis (OR = 0.847, 95% CI = 0.735–0.976, *p* = 0.022). In bidirectional MR, periodontitis was not causally related to any of the cytokines in our study.

**Conclusion:** Our findings provided evidence in support of potential causal associations between circulating IL9/IL17 and periodontitis.

## 1 Introduction

Periodontitis is the sixth most prevalent disease, and severe periodontitis affecting 10.8% of the world’s population (Peres et al., 2019). Major characteristics of periodontitis include destruction of periodontal soft tissues and resorption of alveolar bone, which eventually lead to tooth loss (Hienz et al., 2015). Disruption of oral microbial homeostasis is a major cause for periodontitis (Valm, 2019). In addition, genetic variance, dietary habits, and other diseases also contribute to the development of periodontitis. Identifications of risk factors at the early stage of periodontitis and taking effective intervention strategies accordingly may help to reduce periodontitis-associated tooth loss.

Cytokines play a key role in the development of periodontitis and influence the progression of periodontitis, and are risk factors. Under pathological stimuli, the periodontium stages immune responses that are characterized by accumulation of immune cells and production of cytokines (Cekici et al., 2014). In barrier tissues such as intestine, oral cavity, and skin, cytokines in response to pathological stimuli can become a bridge between parenchymal and non-parenchymal cells, producing an immediate immune response (Kwiecien et al., 2019). At the same time, tissue-resident immune cells and migrating immune cells secrete different types of cytokines in a large amount to participate in the immune response. The cytokines, in turn, exert regulatory effects on each other and are critical for the progression of inflammation. Due to continuous stimulation and damage to the periodontal tissue caused by the pathogenic oral flora and mastication, immune cells such as macrophages and T cells are recruited, and cytokines such as interleukin (IL) 1, IL6 and tumor necrosis factor (TNF) are released in large quantities (Pan et al., 2019). These cytokines can activate the corresponding signaling pathways and further aggravate inflammatory response in periodontal tissue. For example, IL1 activates the nuclear factor kappa-B ligand (RANKL) pathway to promote osteoclast formation, resulting in resorption and destruction of alveolar bone (Huynh et al., 2017).

Previous studies have demonstrated important roles of cytokines and related pathways in the progression of periodontitis. For example, TNF-α inhibits osteoblast differentiation through inhibition of insulin-like growth factor-1 (IGF1), osterix and the runt-related transcription factor-2 (Runx2) (Osta et al., 2014). *In vitro* experiments have shown that IL17 can regulate autophagy and osteoclastogenesis through Beclin1/TAB3/p-ERK pathway (Zhong et al., 2022). However, the findings of these studies were conducted in observational studies that assumed no confounding effects, and therefore could be limited by selection bias, other related diseases, and heterogeneity of comparison groups (Andrews et al., 2020).

Several studies have investigated the relationship between cytokines and periodontitis using meta-analysis. For example, a meta-analysis has included 21 case-control studies to explore the association between IL6 polymorphism and periodontitis susceptibility (Zhu et al., 2016). Another meta-analysis has included 53 studies to investigate the role of multiple cytokine gene polymorphisms in the pathogenesis of periodontitis (Nikolopoulos et al., 2008). However, significant heterogeneity and potential publication bias may occur when analyzing genetic polymorphism of various cytokines from different databases (Liu and Li, 2021). Therefore, evidence for a causal link between cytokines and periodontitis is still lacking.

To assess the causal relationship between exposure and outcome, Mendelian randomization (MR) uses genetic variation (single nucleotide polymorphisms, SNPs) as instrumental variables (IV). At conception, genetic variants are randomly assigned, similar to randomized controlled trials, reducing the effect of confounding factors. Genetic variation was only related to exposure, not outcome, and genetic variation affected outcome only through exposure, not through other pathways (Lawlor et al., 2008). Recently, MR methods have been applied to investigate the genetic relationship between cytokines and the danger of cancer (Bouras et al., 2022), and the causal association between cytokines and risk of multiple sclerosis has also identified by the MR approach (Lu et al., 2021).

In this study, we investigated the genetic causal association between 41 cytokines and periodontitis by applying two sample bidirectional MR, aimed to provide a new perspective on the prevention, diagnosis and treatment of periodontitis.

## 2 Materials and methods

### 2.1 Overall study design

**FIGURE 1:**
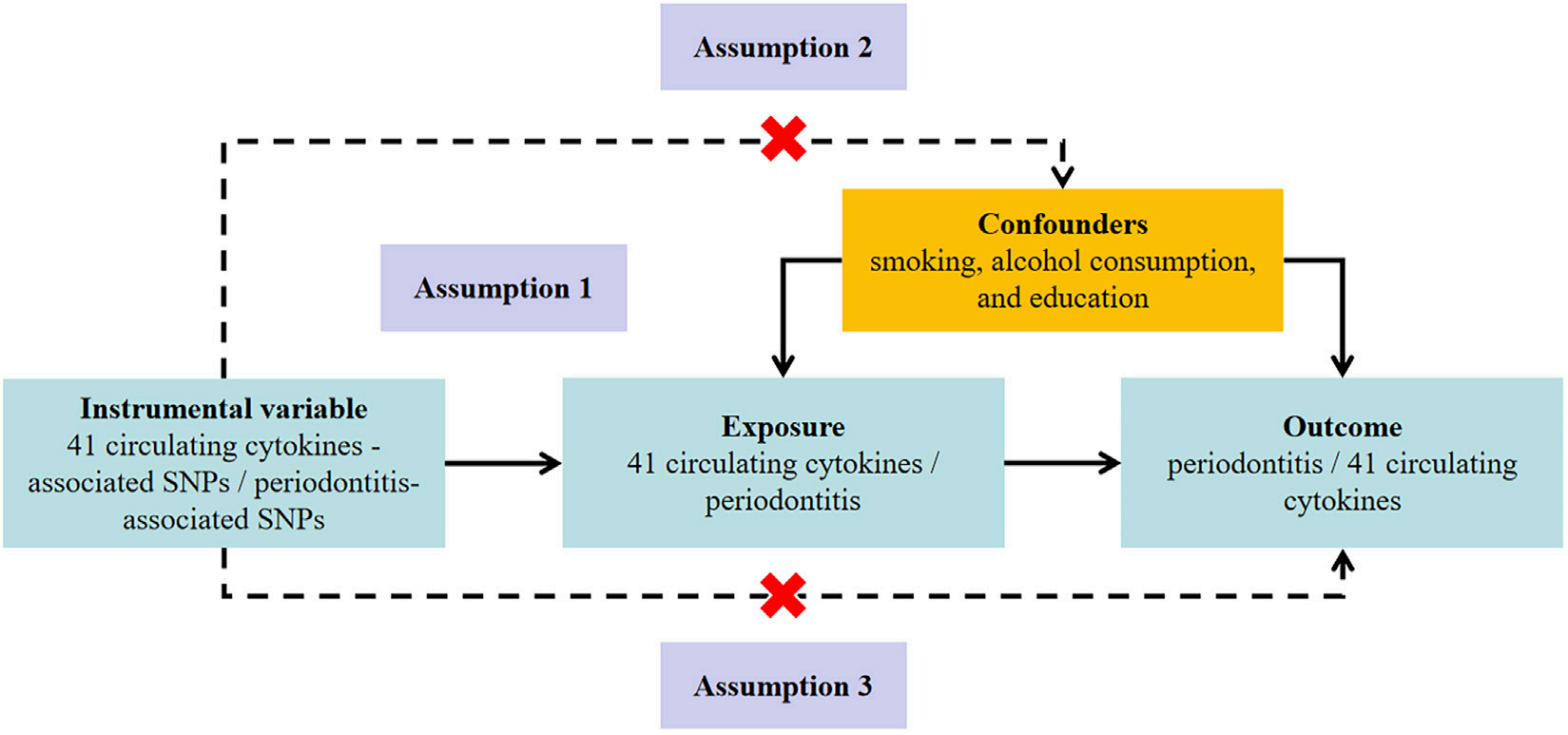
Study design to investigate the association between circulating cytokines and risk of periodontitis basing on bidirectional Mendelian randomization assumptions. The MR study must satisfy three assumptions. Assumption 1: genetic variation used for instrumental variable is associated with exposure; Assumption 2: genetic variation is independent of confounding factors; Assumption 3: genetic variation affects the outcome only through the exposure and not through other pathways. MR, Mendelian randomization; SNP, single-nucleotide polymorphism.

This bidirectional MR study was undertaken following a framework as delineated in Figure 1. The MR approach was based on three assumptions: 1) genetic variation used for IV was associated with exposure; 2) genetic variation was independent of confounding factors; 3) genetic variation affected the outcome only through the exposure and not through other pathways (Lawlor et al., 2008).

### 2.2 Data sources

#### 2.2.1 Summary statistics for cytokines

The summary level datasets of cytokines were obtained from the most recent GWAS, which included 8,293 Finnish individuals from the Cardiovascular Risk in Young Finns Study (YFS) and the FINRISK studies (including “FINRISK 2002” and “FINRISK 1997”) (Ahola-Olli et al., 2017). The cytokine quantification was performed from EDTA treated plasma in FINRISK 1997, heparinized plasma in FINRISK 2002, and sera in YFS. The genetic associations were adjusted for age, sex, body mass index and the first 10 genetic principal components with genomic-control correction. In YFS and FINRISK 2002, a total of 48 cytokines were measured using Bio-Rad’s premixed Bio-Plex Pro Human Cytokine 27-plex Assay and 21-plex Assay, and Bio-Plex 200 reader with Bio-Plex 6.0 software. Only measures within the cytokine-specific detection range were included in the analyses. Cytokines with >90% of values missing were excluded (7 out of 48). In FINRISK 1997, a total of 17 cytokines overlapped with those measured in FINRISK2002 and YFS and were thus included in the GWAS (Ahola-Olli et al., 2017).

#### 2.2.2 Summary statistics for periodontitis

Summary statistics for periodontitis were obtained from the latest meta-analysis of the largest sample size to date for the GWAS (GLIDE) consortium of dental endpoints involved 17,353 clinically diagnosed cases and 28,210 controls (Shungin et al., 2019). After excluding the Hispanic Community Health Study/Latino Study (HCHS/SOL) in the GLIDE consortium, there were 12,289 cases and 22,326 controls of European ancestry. Clinical diagnostic criteria from the Centers for Disease Control and Prevention/American Academy of Periodontology and self-reported diagnoses from the Brigham and Women’s Hospital Women’s Health Study were primarily used, while additional inclusion criteria were defined as one of the following: periodontal probing depth ≥5 mm on two or more tooth surfaces, or ≥4 mm on four or more tooth surfaces, ≥5.5 mm on two or more tooth surfaces or “gum surgery” from dental records (Shungin et al., 2019).

### 2.3 Selection of genetic variants as IVs

To ensure the veracity and accuracy of the conclusions regarding the causal relationship between cytokines and periodontitis risk, the following quality control steps were used to select the best genetic tool. First, a set of SNPs below the genome-wide threshold of statistical significance (5 × 10^−8^) was used as IV, since only 26 SNPs for cytokines reached genome-wide significance. To obtain more comprehensive results, we also selected another set of SNPs below the genome-wide significance level (5 × 10^−6^) as IVs. Second, the PLINK clustering method (*R* ^2^ < 0.001, clustering distance = 10,000 kb) was used to assess the linkage disequilibrium (LD) among the included SNPs. Third, SNPs for cervical cancer will not be included in the instrumental variables. Fourth, the F-statistic = *R* ^2^ × (N-2)/(1-R^2^) was calculated for each IV used for cytokines as a measure of instrumental strength, where *R* ^2^ is the phenotypic variation explained by each genetic variant in the exposure and N is the sample size (Palmer et al., 2012). Generally, F-statistics >10 was set as the threshold of strong IV (Burgess et al., 2017). Finally, when exposure-related SNPs were not present in the resultant GWAS, proxy SNPs significantly associated with the variant of interest were selected (*r* ^2^ > 0.8). Ultimately in our analysis, 8 SNPs (*p* < 5 × 10^−6^) were used as IVs in periodontitis. 1 to 6 SNPs (*p* < 5 × 10^−8^) and 4 to 16 SNPs (*p* < 5 × 10^−6^) were used as IVs in cytokines. The detailed information on the IVs was displayed in Supplementary Tables S1–S3.

### 2.5 Statistical analysis

To evaluate the genetic causal effects, several different methods were applied, which provided valid evidence under different conditions, with Inverse variance weighted (IVW) serving as the primary outcome (Burgess et al., 2017). In order to aggregate the Wald values for individual SNP and generate an estimated total value of the impact, a meta-analysis methodology was used in the IVW model. In the absence of horizontal pleiotropy and heterogeneity, IVW linear regression could provide an unbiased causal estimate (Burgess et al., 2017). For traits with only one SNP, the Wald ratio test was applied. By assuming that exposure and result have a linear relationship and that the estimates of the genetic association have a normal bivariate distribution, the Maximum likelihood method (ML) generates estimated causal associations (Milligan, 2003). MR-Egger can offer a reliable, unbiased estimate even if all of the SNPs in the selection are invalid (Burgess and Thompson, 2017). Although as much as 50% of the data used in the study comes from variations of interest that are invalid IVs, the weighted median can still produce reliable estimates of the causal effects (Bowden et al., 2016a). When the causal estimates of most instrumental variables are similar, the weighted model approach remains valid even if other instrumental variables do not qualify for the causal inference of the MR technique (Hartwig et al., 2017). The Robust Adjusted Profile Score (MR-RAPS) is statistically robust to weak instruments and robust in the presence of systematic pleiotropy. MR-RAPS can lessen horizontal pleiotropy by taking into account the measurement error in the association of SNPs and exposure (Zhao et al., 2019).

The Cochran Q statistic and I^2^ statistic were used to test the heterogeneity. Random-effect IVW is employed if there is heterogeneity among the SNPs included in each analysis (Bowden et al., 2016b). To determine if a specific genetic locus had an impact on random estimates, the leave-one-out sensitivity method was used. Additionally, scatterplots and funnel plots were created for further analysis. The requirement that exposure-related SNPs exclusively affect the outcome through themselves is crucial for the prerequisite of the MR method. To determine whether IV increases the degree of polymorphic impact of the results through more than one biological pathway, we tested for polymorphism using MR-Egger regression and Mendelian randomized polymorphism RESidual Sum and Outlier (MR-PRESSO) (Verbanck et al., 2018). Furthermore, we searched all SNPs in the PhenoScanner database (Kamat et al., 2019) to evaluate whether those SNPs were significantly associated (*p* < 5 × 10^−8^) with periodontal disease risk factors, including smoking, alcohol consumption, and education. The causal associations were re-estimated after removing pleiotropic SNPs in sensitivity analyses to avoid potential pleiotropic effects.

The threshold for statistical significance for the association between cytokines and periodontitis was set to 1.2 × 10^−3^ (*p* = 0.05/41) using a Bonferroni-adjusted *p*-value. If *p* < 0.05 but above the Bonferroni-adjusted significance threshold, this was considered suggestive of evidence for a potential association. All the analyses and relevant figures were made by R 4.1.2 (R Foundation for Statistical Computing, Vienna, Austria), using the “TwoSampleMR (0.5.6)”, “MR-PRESSO (1.0)”, “mr.raps (0.2)”, and “ggforestplot (0.1.0)” packages. Reporting follows the STROBE-MR statement (Skrivankova et al., 2021). The study protocol was not pre-registered. Power calculations were performed in mRnd, a Web-based application (https://shiny.cnsgenomics.com/mRnd/) (Brion et al., 2013) assuming a 5% type I error rate. Statistical significance was set to a two-sided *α* of 0.05.

## 3 Results

### 3.1 Characteristics of the selected SNPs

Only 26 among the 41 cytokines have independent genome-wide significant SNP when the cut-off is set to *p* < 5 × 10^−8^, whereas all 41 have independent SNPs when using a higher cut-off (*p* < 5 × 10^−6^) (Supplementary Figure S1, Figure ). At genome-wide significance, 1 to 6 independent genome-wide significant SNPs are used for cytokines explained 0.36%–19.48% of the variance in cytokines, corresponding to F-statistic of 29.98–1731.64 (Supplementary Table S1). In the higher cut-off value (*p* < 5 × 10^−6^), 4 to 16 SNPs are used as IVs in cytokines, and 8 SNPs are used as IVs in periodontitis. The genetic variance they explained are 0.86%–22.99% and 0.53%, corresponding to F-statistic of 71.44–2023.73 and 44.23 respectively (Supplementary Table S2, Supplementary Table S3). In the PhenoScanner search, we find that both rs3748034 and rs7088799 are associated with alcohol consumption and education, so we re-analyze after eliminating these SNPs (Supplementary Table S4). In the periodontitis GWAS the targeted SNPs are not found, rs138987090 and rs78422482 were replaced by proxy SNP rs149524246 and rs115349882 with *R* ^2^ > 0.8 respectively. Details about identified SNPs are presented in Supplementary Tables S1–S3 **.**

**FIGURE 2:**
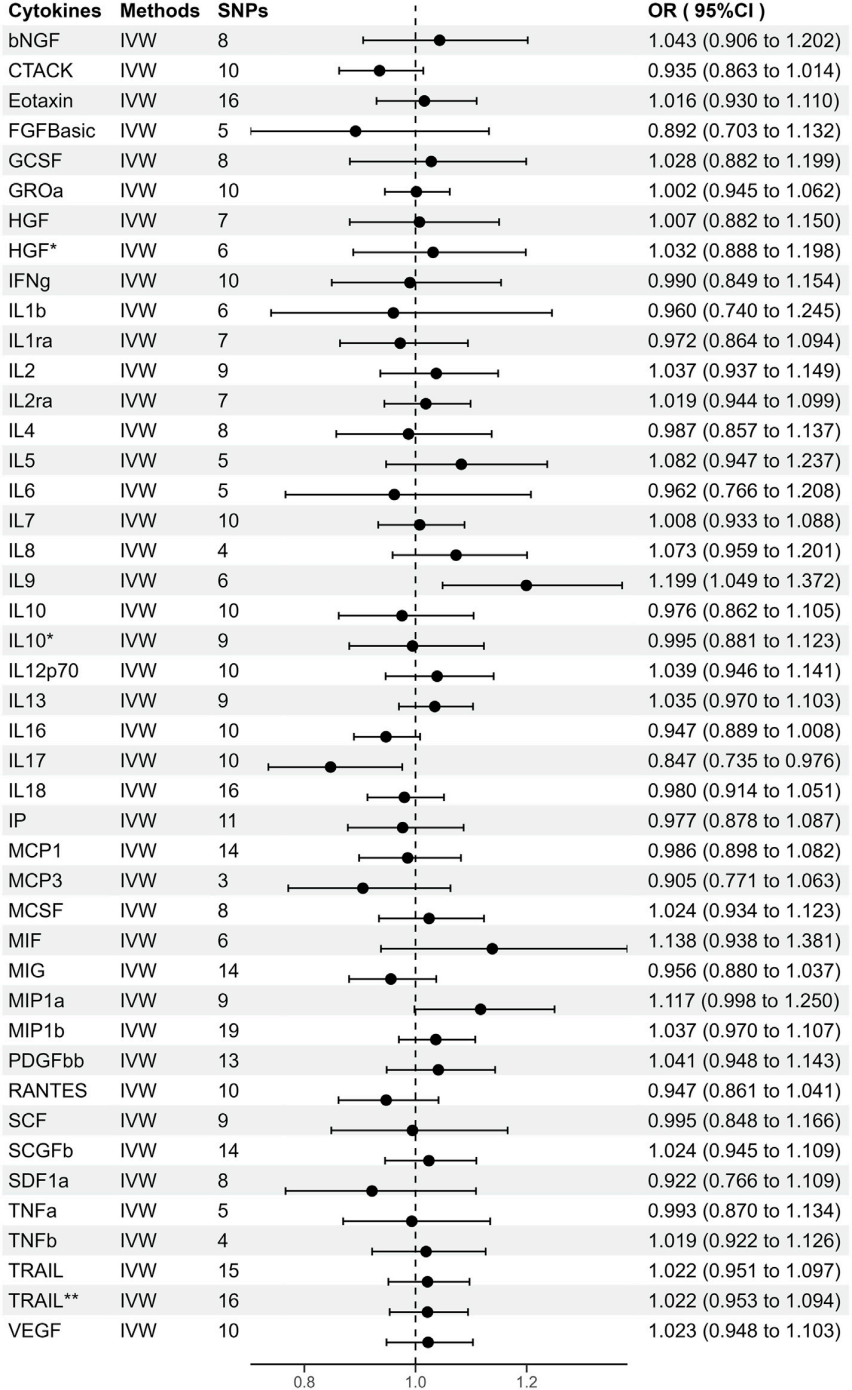
Odds ratios (95% Confidence interval) from Mendelian randomization analysis showing associations between genetically predicted cytokine levels (cut-off = *p* < 5 × 10^−6^) and risks for periodontitis. The causal association between circulating cytokine levels and periodontitis was mainly calculated through two-sample MR analysis using IVW method. Estimates with 95% CI are expressed as odds ratios for periodontitis per 1-SD increase of genetically predicted circulating cytokine levels. *, Results of MR analysis after exclusion of SNPs associated with confounding factors. **, Results of MR analysis after using a proxy SNP.

### 3.2 Statistical power to detect effect sizes

*A priori* statistical power is calculated (Brion et al., 2013). Given *α* = 5%, we had ≥80% power for the primary analysis when the expected odds ratios (ORs) for periodontitis are ≥1.38 or ≤0.71 for 41 circulating cytokines. Detailed information on the power calculation is included in Supplementary Table S5.

### 3.3 Genetic prediction of circulating cytokines for periodontitis risk

**FIGURE 3:**
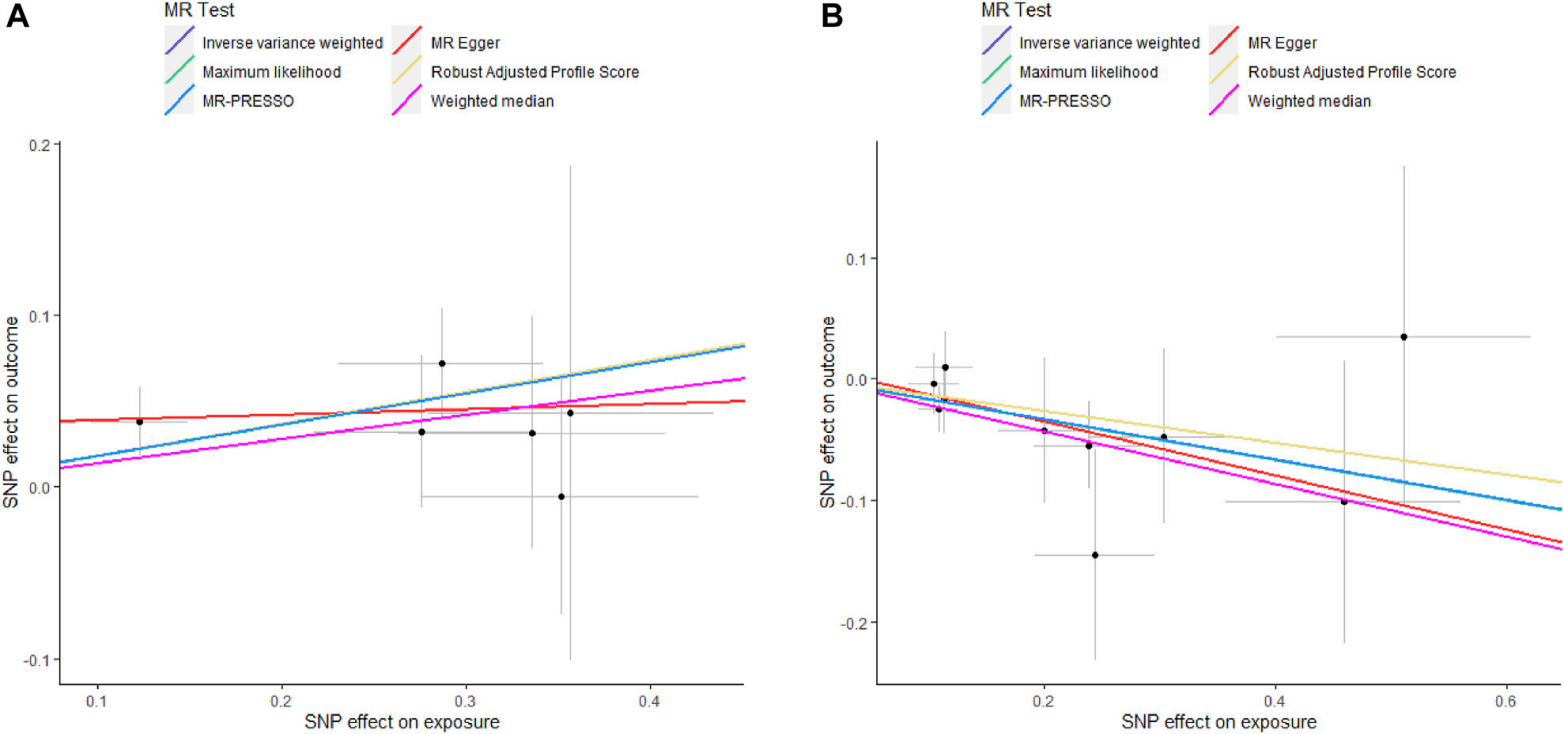
Scatter plots of potential effects of SNPs on IL9 and IL17 *versus* periodontitis. A, Scatter plot of potential effects of SNPs on IL9 *versus* periodontitis. B, Scatter plot of potential effects of SNPs on IL17 *versus* periodontitis. Scatter plots presented per-allele association with outcome risk plotted against the per-allele association with one standard deviation of exposure (with vertical and horizontal gray lines showing the 95% CI for each SNP). Analyses were conducted using IVW, Weighted median, Wald ratio, RAPS, MR Egger, MR-PRESSO, and ML. The slope of each line corresponding to the estimated MR effect per method. SNP, single-nucleotide polymorphism; IVW, Inverse variance weighted; ML, Maximum likelihood; MR-PRESSO, Mendelian Randomization Pleiotropy RESidual Sum and Outlier; RAPS, Robust Adjusted Profile Score.

As shown in Supplementary Figure S1, genetically predicted circulating cytokines are not significantly associated with periodontitis. When using SNPs obtained with the higher cut-off (*p* < 5 × 10^−6^), the MR study reveals that genetically predicted circulating IL9 is potentially associated with periodontitis (OR = 1.199, 95%CI: 1.049–1.372 *p* = 0.008) based on the IVW method (Table 1). The Maximum likelihood, MR-RAPS, and MR-PRESSO method also provide similar results. Furthermore, based on the IVW method, the MR analysis show that there is a potential negative correlation between genetically predicted circulating IL17 and Periodontitis (OR = 0.847, 95%CI: 0.735–0.976, *p* = 0.022) (Table 1). The similar results are also obtained in the Maximum likelihood, the weighted median, and MR-PRESSO methods. The results are shown in Table 1 and Figure 2. Cochran Q statistic and I^2^ statistic indicate low heterogeneity and more reliability of these SNPs (all *p* > 0.05) (Table 1). The MR-Egger intercept suggests that stem cell factor (SCF) predicted by SNPs (*p* < 5 × 10^−6^) might be pleiotropic SNPs, but MR-PRESSO global test does not identify any pleiotropic SNPs. Beyond that, there is no evidence of horizontal pleiotropy for SNPs. Detailed information about the results is included in Supplementary Tables S6–S7. The estimated effect sizes for SNPs of cytokines on periodontitis are displayed in scatter plot (Figure 3). The funnel plots show general symmetry, suggesting little evidence of heterogeneity (Supplementary Figure S2). Leave-one-out sensitivity analyses suggest that there is no individual SNP with a strong influence on the overall effect (Supplementary Figure S3).

**TABLE 1:** Estimation of associations between circulating IL9, IL17 and risk of periodontitis using Mendelian randomization analysis.

		MR results					Heterogeneity test				Horizontal pleiotropy test				
							Cochrane Q test				MR-egger intercept test			MR-PRESSO global test	
Methods		SNPs	Beta	SE	*P*	Or (95% CI)	Q	df	I^2^	*P*	Intercept	SE	*P*	RSSobs	*P*
IL9	IVW	6	0.182	0.069	0.008	1.199 (1.049–1.372)	2.376	5	0	0.795	−0.053	0.029	0.106	3.700	0.788
	ML	6	0.183	0.070	0.009	1.201 (1.047–1.378)	2.254	5	0	0.813					
	MR Egger	6	0.031	0.169	0.862	1.032 (0.741–1.438)	1.431	4	0	0.839					
	Weighted median	6	0.141	0.091	0.124	1.151 (0.962–1.377)									
	RAPS	6	0.185	0.061	0.002	1.203 (1.068–1.356)									
	MR-PRESSO	6	0.182	0.047	0.012	1.199 (1.093–1.316)									
IL17	IVW	10	−0.166	0.072	0.022	0.847 (0.735–0.976)	3.794	9	0	0.924	0.009	0.025	0.716	4.450	0.939
	ML	10	−0.167	0.074	0.023	0.846 (0.733–0.978)	3.733	9	0	0.928					
	MR Egger	10	−0.222	0.166	0.218	0.801 (0.578–1.109)	3.652	8	0	0.887					
	Weighted median	10	−0.217	0.090	0.016	0.805 (0.675–0.961)									
	RAPS	10	−0.131	0.073	0.070	0.877 (0.761–1.011)									
	MR-PRESSO	10	−0.166	0.047	0.006	0.847 (0.773–0.929)									

Estimates with 95% CI, are expressed as odds ratios for periodontitis per 1-SD, increase in genetically predicted circulating IL9 and IL17 levels. Cochran Q-test was used to assess heterogeneity between SNP-specific estimates, and MR-Egger regression and MR-PRESSO, were used to test for evidence of polymorphism. IL, interleukin; SNP, single-nucleotide polymorphism; SE, standard error; OR, odds ratios; CI, confidence interval; df, degree of freedom; RSSobs, observed residual sum of squares; IVW: inverse variance weighted; ML: maximum likelihood; RAPS, robust adjusted profile score; MR-PRESSO, Mendelian randomization Pleiotropy RESidual Sum and Outlier.

### 3.4 Genetic prediction of periodontitis for risk of circulating cytokines

**FIGURE 4:**
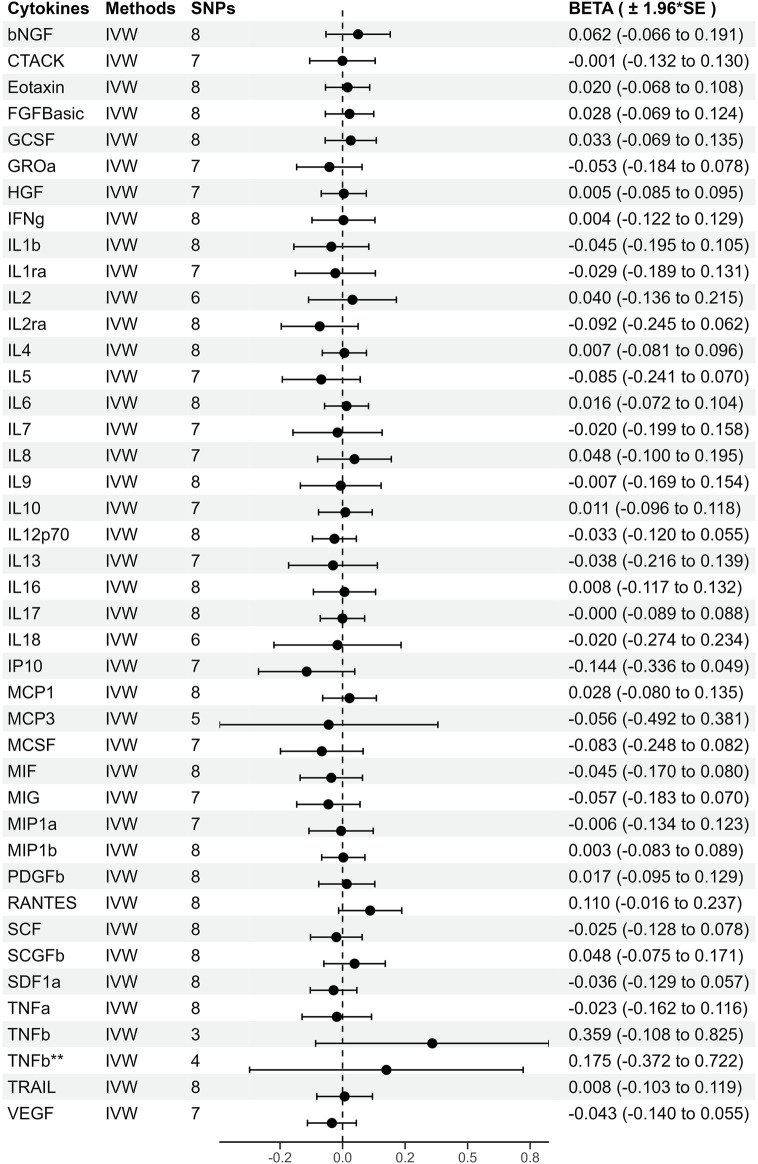
Beta (±1.96*Standard error) from Mendelian randomization analysis showing associations between genetically predicted periodontitis (Cut-off = *p* < 5 × 10^−6^) and risks for cytokine levels. The causal association between periodontitis and circulating cytokine levels was mainly calculated through two-sample MR analysis using IVW method. *, Results of MR analysis after exclusion of SNPs associated with confounding factors. **, Results of MR analysis after using a proxy SNP.

As shown in Figure 4, genetically predicted periodontitis is not significantly associated with any of the 41 circulating cytokines based on any method. MR-PRESSO and the MR-Egger intercept do not identify any pleiotropic SNP. Except for IL1b as outcome, Cochran Q statistic and I^2^ statistic indicate low heterogeneity and more reliability of these SNPs (all *p* > 0.05). Since the presence of heterogeneity, we adopt the results of the random-effect IVW effects model, which does not identify causal relationship between genetically predicted periodontitis and IL1b. Detailed information about the results is included in Supplementary Table S1.

## 4 Discussions

In this study, we use MR analysis to investigate the potential causal relationship between the genetic basis of circulating levels of 41 cytokines and the risk of periodontitis. We find that genetic proxies of circulating concentration of IL9 are positively associated with the risk of periodontitis, and those of IL17 are negatively with the risk of periodontitis.

IL9 exhibits pro-inflammatory activities in a range of diseases, but its role in periodontitis is unclear. Numerous studies have indicated the involvement of IL9 in the pathogenesis of allergic diseases. In addition, IL9 promotes tumorigenesis through autocrine and anti-apoptotic activity (Feng et al., 2011). A recent study has demonstrated that IL9 plays a central role in osteoclast genesis by enhancing M-CSF/sRANKL-mediated osteoclast formation and function (Kar et al., 2021). Our study reveals a genetic link between IL9 and periodontitis. Using MR approach, we show that elevated circulating concentrations of IL9 predispose individuals to the development of periodontitis. A previous meta-analysis assessing genetic polymorphisms of IL9 in Chinese non-smokers has suggested that IL9 does not have a causal relationship with periodontitis (Zheng et al., 2020). We use MR to analyze the latest GWAS meta-analysis of the GLIDE Consortium, the largest sample size to date involving 17,353 clinically diagnosed cases and 28,210 controls (Shungin et al., 2019). Consistent with our results, a positive association between IL9 and the severity of periodontal disease has been established by assessing IL9 levels in gingival sulcus fluid samples and biopsies (Díaz-Zúñiga et al., 2017). Although IL9 has been understudied in the field of periodontitis, our study provides a genetic basis for a causal relationship between IL9 and periodontitis. Future studies are warranted to understand the regulation of IL9 in periodontitis in more details, to identify relevant target cells of IL9, and to delineate the mechanisms that mediate the effects of IL9 in periodontitis.

Another interesting finding of our study is an inverse association between genetically proxied circulating IL17 concentrations and periodontitis using the MR approach. IL17 is a widely reported pro-inflammatory cytokine involved in the pathogenesis inflammatory diseases and autoimmune diseases (McGeachy et al., 2019). IL17 also induces an increase in osteoclasts not only by stimulating the secretion of IL6 and RANKL, but also by altering the microbial composition of the oral cavity, increasing levels of Enterobacteriaceae, *Enterococcus* and *Staphylococcus*, which can aggravate periodontitis (Xiao et al., 2017). Our findings were in the opposite direction of the expected association. Supporting our results, deficiency of IL17 receptor A has been demonstrated to accelerate bone loss decreased neutrophil accumulation to the periodontal lesions in an experimental periodontitis model, indicating a protective effect of IL17 (Yu et al., 2007). Moreover, IL17 receptor A signaling plays a protective role in periapical bone destruction stimulated by infection (AlShwaimi et al., 2013). Using meta-analysis, polymorphisms of IL17F gene rs763780 and IL17A gene rs2275913 have recently been detected in patients with periodontitis, but there was no significant association between IL17 and periodontitis (Mazurek-Mochol et al., 2021). It is clear that IL17 has pleiotropic functions in the immune system (McGeachy et al., 2019), likely contributing to its different roles in different diseases. More studies are needed to precisely understand the mechanism of IL17 in periodontitis.

The main strengths of this study included the following. First, we used several MR techniques, including bidirectional MR to detect direction of association, PhenoScanner to exclude confounders, and sensitivity analyses to enhance the robustness of the results. Second, our study included a total of 41 cytokines from multiple classes, which permitted an examination of the wider inflammatory network in periodontitis. Third, our GWASs of exposure and outcome were the largest sample size to date, which added statistical power, and to minimize the risk of bias due to sample overlap, the GWAS of exposure and result came from entirely different consortia.

However, this study has potential limitations. First, we used a higher *p* < 5 × 10^−6^ cut-off for obtaining relatively abundant SNPs predicting the circulating cytokines. This could increase the risk of weak instrument bias for individual genetic variants, although our F-statistics suggested that these instruments had appropriate strength. Second, the potential synergistic effects between the studied cytokine networks were not taken into account. Third, our findings did not meet the strict multiple test threshold. It is necessary to test these findings with larger GWAS. Future MR studies using single- or multi-trait GWAS with larger number of cytokines may be able to address some of these issues.

## 5 Conclusion

As inflammatory regulators, cytokines are important risk factors for inflammatory diseases, and they can be exploited in GWAS to elucidate biochemical pathways underlying the link from locus to disease susceptibility. In our study, IL9 and IL17 were found to be genetically associated with periodontitis. These genetic associations may contribute to the understanding of pathological mechanisms, and provide innovative approaches for risk assessment, outcome prediction, and treatment for periodontitis.

## Data Availability

The original contributions presented in the study are included in the article/Supplementary Material, further inquiries can be directed to the corresponding authors.
