# Supplementary material for: Assessment of bidirectional relationships between circulating cytokines and periodontitis: Insights from a mendelian randomization analysis
Source: Front Genet. 2023 Jan 30;14:1124638. doi: 10.3389/fgene.2023.1124638 (PMC9923016; doi:10.3389/fgene.2023.1124638)
Supplement: Supplementary file 2 [file Image1.pdf]

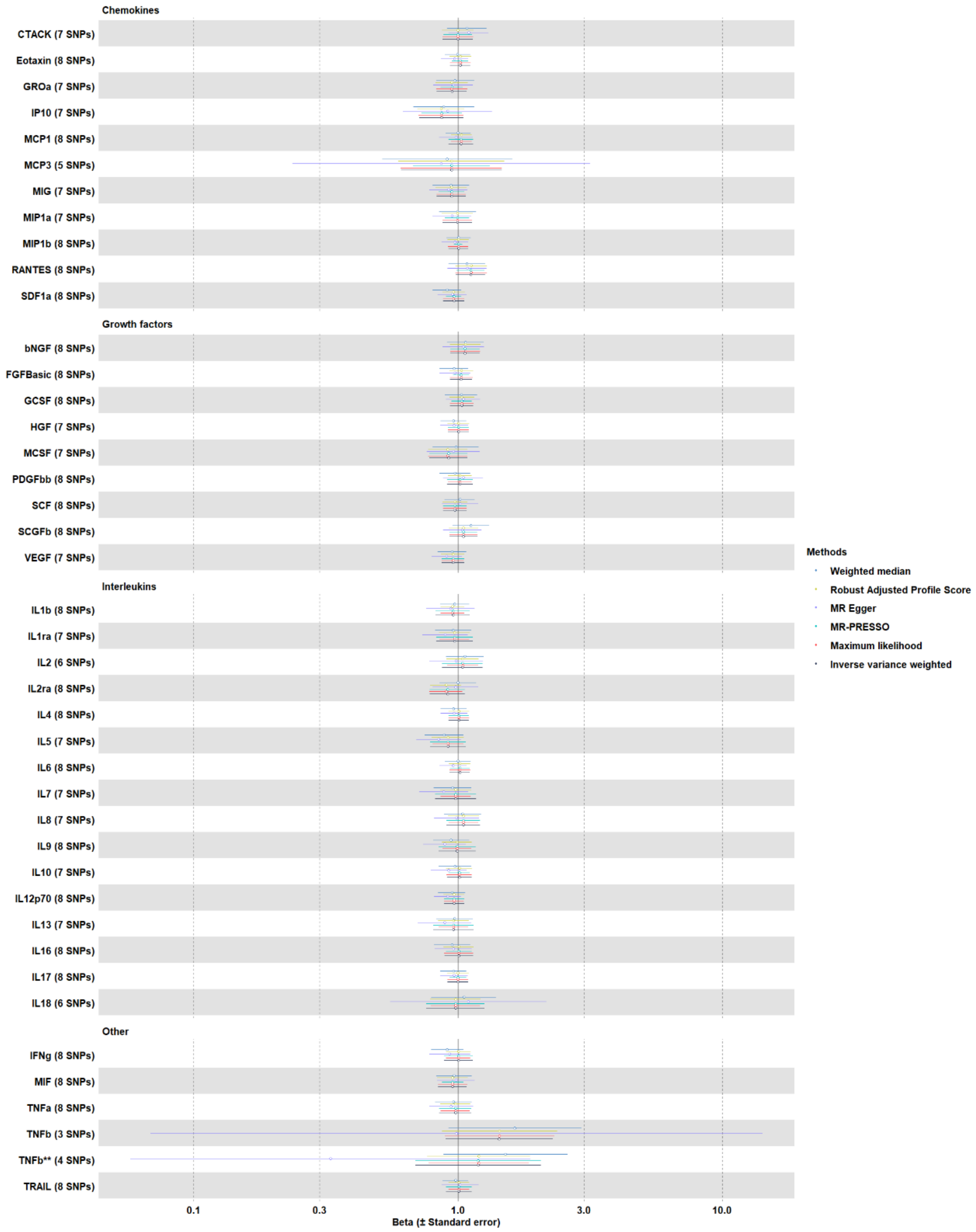

Figure S1 Beta (± Standard error) from Mendelian Randomization analysis showing associations between genetically predicted periodontitis (Cut-off=$P<5\times10^{-6}$) and risks for cytokine levels

The causal association between periodontitis and circulating cytokine levels was mainly calculated through two-sample MR analysis using IVW method. Weighted median, Wald ratio, RAPS, MR Egger, MR-PRESSO, and ML methods were used to test the robustness of results from IVW method. *, Results of MR analysis after exclusion of SNPs associated with confounding factors. **, Results of MR analysis after using a proxy SNP.

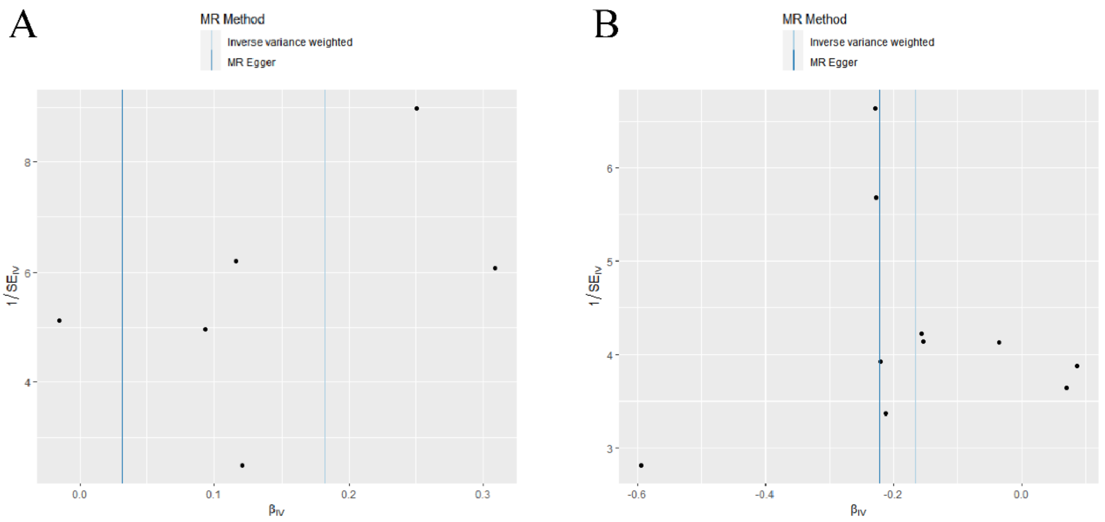

Figure S2 Funnel plots for Mendelian Randomization analyses of the causal effect of IL9, IL17 on periodontitis.

A, Funnel plot for MR analyses of the causal effect of IL9 on Periodontitis. B, Funnel plot for MR analyses of the causal effect of IL17 on periodontitis. IVW and MR Egger methods were used to detect the heterogeneity of SNP. The funnel plots showed general symmetry, suggesting little evidence of heterogeneity. MR, Mendelian Randomization; IVW, Inverse variance weighted; SNP, single-nucleotide polymorphism.

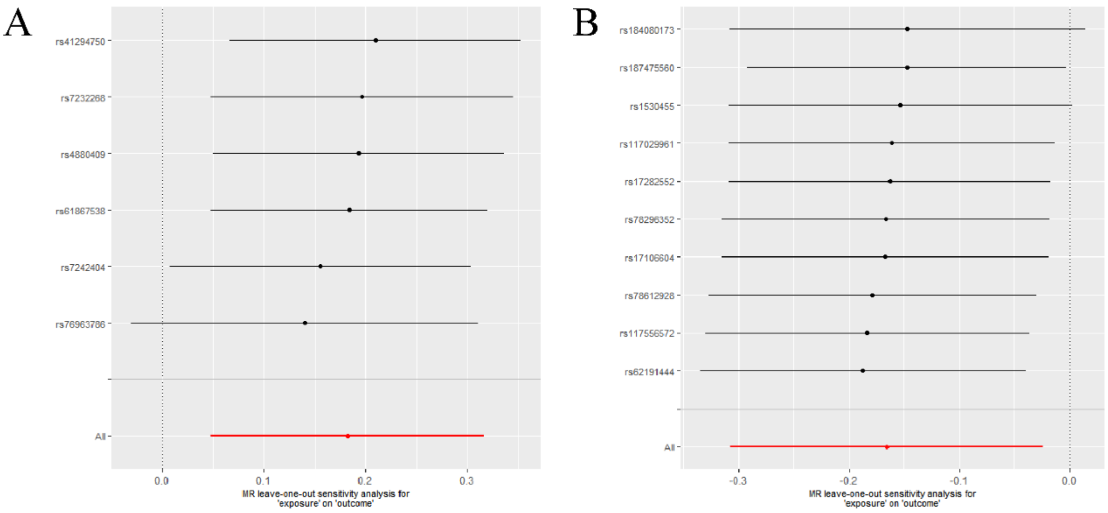

Figure S3 Leave-one-out sensitivity based on IVW model for IL9, IL17 on periodontitis.

A, Leave-one-out sensitivity based on IVW model for IL9 on periodontitis. B, Leave-one-out sensitivity based on IVW model for IL17 on periodontitis. The overall estimate (red horizontal line) wasnot affected by the removal of a single variable (black horizontal line). There was no evidence of obvious heterogeneity, indicating that no specific SNP alone accounted for the association between IL9/IL17 and periodontitis. The results suggested that there was no individual SNP with a strong influence on the overall effect. SNP, single-nucleotide polymorphism; IVW, Inverse variance weighted.
